# Phase I study of high-dose epirubicin and vinorelbine in previously untreated non-small-cell lung cancer stage IIIB-IV.

**DOI:** 10.1038/bjc.1995.545

**Published:** 1995-12

**Authors:** M. Bakker, H. J. Groen, E. F. Smit, J. Smeets, M. Riggi, P. E. Postmus

**Affiliations:** Department of Pulmonary Diseases, University Hospital, Groningen, The Netherlands.

## Abstract

The aim of the study was to determine the maximum tolerated dose (MTD) for the combination of high-dose epirubicin and vinorelbine in chemotherapy-naive patients with inoperable non-small-cell lung cancer (NSCLC). Twenty-one patients with stage IIIB and IV NSCLC were treated in a single-centre study with escalating doses of epirubicin and vinorelbine given on an outpatient basis. The first dose level comprised epirubicin 100 mg m-2 on day 1 and vinorelbine 20 mg m-2 (days 1 and 8) given intravenously every 3 weeks. Escalating doses for epirubicin and vinorelbine were respectively 120 (day 1) and 20 (days 1 and 8), 120 (day 1) and 25 (days 1 and 8) and 135 (day 1) and 25 (days 1 and 8) mg m-2. Inclusion criteria were age < or = 75 years, ECOG performance score < or = 2 and normal renal, hepatic and bone marrow functions. Dose-limiting toxicities were thrombocytopenia grade II and neutropenia grade III on day 8, febrile neutropenia, and neutropenia lasting > 7 days. No dose-limiting toxicity (DLT) was observed at the first dose level; at the 135/25 mg m-2 dose level three out of six patients had a DLT which was considered as unacceptable. The only non-haematological toxicity reaching grade III was nausea/vomiting. One patient showed cardiac toxicity. No neurotoxicity and no treatment-related deaths were seen. The maximum tolerated dose of epirubicin and vinorelbine is 135 mg m-2 (day 1) and 25 mg m-2 (days 1 and 8) respectively, causing mainly haematological toxicity. The recommended dose of epirubicin and vinorelbine for phase II studies is found to be 120 mg m-2 and 20 mg m-2 respectively.


British Journal of Cancer (1995) 72, 1547–1550
© 1995 Stockton Press All rights reserved 0007-0920/95 $12.00

### Phase I study of high-dose epirubicin and vinorelbine in previously untreated non-small-cell lung cancer stage IIIB–IV

M Bakker[1], HJM Groen[1], EF Smit[1], J Smeets[2], M Riggi[2] and PE Postmus[3]

[1]*Department of Pulmonary Diseases, University Hospital, Groningen, The Netherlands;* [2]*Medical Department, Pharmacia, The Netherlands and Italy;* [3]*Department of Pulmonary Diseases, Free University Hospital, Amsterdam, The Netherlands*

**Summary** The aim of the study was to determine the maximum tolerated dose (MTD) for the combination of high-dose epirubicin and vinorelbine in chemotherapy-naive patients with inoperable non-small-cell lung cancer (NSCLC). Twenty-one patients with stage IIIB and IV NSCLC were treated in a single-centre study with escalating doses of epirubicin and vinorelbine given on an outpatient basis. The first dose level comprised epirubicin 100 mg $m^{-2}$ on day 1 and vinorelbine 20 mg $m^{-2}$ (days 1 and 8) given intravenously every 3 weeks. Escalating doses for epirubicin and vinorelbine were respectively 120 (day 1) and 20 (days 1 and 8), 120 (day 1) and 25 (days 1 and 8) and 135 (day 1) and 25 (days 1 and 8) mg $m^{-2}$. Inclusion criteria were age $\leqslant 75$ years, ECOG performance score $\leqslant 2$ and normal renal, hepatic and bone marrow functions. Dose-limiting toxicities were thrombocytopenia grade II and neutropenia grade III on day 8, febrile neutropenia, and neutropenia lasting $>7$ days. No dose-limiting toxicity (DLT) was observed at the first dose level; at the 135/25 mg $m^{-2}$ dose level three out of six patients had a DLT which was considered as unacceptable. The only non-haematological toxicity reaching grade III was nausea/vomiting. One patient showed cardiac toxicity. No neurotoxicity and no treatment-related deaths were seen. The maximum tolerated dose of epirubicin and vinorelbine is 135 mg $m^{-2}$ (day 1) and 25 mg $m^{-2}$ (days 1 and 8) respectively, causing mainly haematological toxicity. The recommended dose of epirubicin and vinorelbine for phase II studies is found to be 120 mg $m^{-2}$ and 20 mg $m^{-2}$ respectively.

**Keywords**: non-small-cell lung cancer; phase I; epirubicin; vinorelbine

Patients with non-small-cell lung cancer (NSCLC) are often diagnosed with locally advanced or disseminated disease despite few or no symptoms. Median survival for patients with advanced locoregional inoperable NSCLC is 8–10 months (Bunn, 1991), for patients with metastatic NSCLC median survival is 6 months (Ihde, 1992). To improve the prognosis of patients with inoperable NSCLC, chemotherapy with or without radiotherapy is the only currently available treatment modality. A meta-analysis of results of (combination) chemotherapy trials in these patients has shown improvement of survival compared with best supportive care only (Souquet *et al.*, 1993), although the benefit at 1 year is only marginal (Grilli *et al.*, 1993). A limited number of cytotoxic drugs induce more than 15% objective responses as single agent in NSCLC. One of these agents is epirubicin, the 4′ epimer of the anthracycline antibiotic doxorubicin (DXR). The major acute dose-limiting toxicity (DLT) of anthracyclines is myelosuppression; the most important chronic DLT is cardiotoxicity which is manifested as irreversible cardiomyopathy (Plosker *et al.*, 1993). In previous comparative studies *vs* DXR, epirubicin has demonstrated less bone marrow and cardiac toxicity at equipotent dosages; its major acute DLT is myelosuppression (Launchbury and Habboubi, 1993). In NSCLC high-dose epirubicin ($>120$ mg $m^{-2}$) as a single agent has shown promising objective response rates of 21–56% (Wils *et al.*, 1990; Martoni *et al.*, 1991; Villar *et al.*, 1991; Feld *et al.*, 1992; Smit *et al.*, 1992). One way to improve these results is to combine high-dose epirubicin with other active agents in NSCLC. Vinorelbine (nor-5′anhydrovinblastine) is a new semisynthetic vinca alkaloid chemically different from vinblastine by a substitution of the catharanthine moiety. Like other vinca alkaloids, vinorelbine inhibits tubulin polymerisation into microtubules, and as in the parent vinblastine, neurotoxicity appears to be mild (Krikorian *et al.*, 1991; Zhou *et al.*, 1992). Vinorelbine has been under investigation in phase II trials and showed activity against NSCLC with response rates ranging from 14% to 65% (Cvitovic *et al.*, 1992; Sorensen, 1992; Lilenbaum *et al.*, 1993). The major toxicities of vinorelbine are myelosuppression and nausea and vomiting. One large multicentre study has shown favourable survival rates for vinorelbine combined with cisplatin compared with single-agent vinorelbine (Le Chevalier *et al.*, 1994). We conducted a phase I study combining high-dose epirubicin with vinorelbine in stage IIIB/IV NSCLC patients.

Correspondence: HJM Groen, Department of Pulmonary Diseases, University Hospital Groningen, Hanzeplein 1, 9713 GZ Groningen, The Netherlands
Received 24 March 1995; revised 3 July 1995; accepted 10 July 1995.

### Materials and methods

### *Patients and staging*

Between March 1992 and January 1994 21 patients were entered into this study. Eligibility criteria were histologically proven NSCLC with disseminated or unresectable disease not amenable to surgery. No prior chemotherapy was allowed. Patients who had received prior radiotherapy had to have finished treatment at least 4 weeks before entry. Additional inclusion criterion were age between 18 and 75 years, Eastern Cooperation Oncology Group (ECOG) performance score (PS)$\leqslant 2$, white blood count (WBC)$\geqslant 4.0 \times 10^9 l^{-1}$, neutrophils$\geqslant 2.0 \times 10^9 l^{-1}$, platelets $\geqslant 100 \times 10^9 l^{-1}$, bilirubin$<$ 35 $\mu$mol $l^{-1}$ and creatinine$<120$ $\mu$mol $l^{-1}$. Patients wtih symptomatic brain metastases, myocardial infarction within the last 12 months, left ventricular ejection fraction (LVEF) less than 90% of lower normal institutional limit as measured by multiple ECG-gated radionuclide study (MUGA scan), arrhythmias requiring permanent medication, uncontrolled hypertension or ischaemic heart disease were ineligible. Approval of the Medical Ethical Committee of the University Hospital in Groningen, The Netherlands, was obtained, and all patients gave written informed consent before entry into the study. Before treatment all patients underwent baseline measurements of bone marrow and cardiac function and staging procedures including physical examination, chest radiograph, computerised tomography (CT) scan of thorax and upper abdomen, ECG and MUGA scan and laboratory tests including complete blood cell counts, electrolytes, liver and renal functions. Additional studies including bone scintigraphy or CT scan of the brain were performed on indication of suspected metastases.

*Toxicity*

Toxicity was measured according to the WHO grading system (WHO, 1978). Complete blood cell counts including WBC and differentials were performed on days 1, 8, 10, 12, 15, 17 and 22 of each course. In the last week of each course an ECG was obtained, and liver and renal functions as well as tumour measurements and toxicity scores were determined. LVEF was measured by MUGA scan after three cycles and/or at discontinuation of therapy. Patients who completed treatment were assessed for chronic toxicity every 2 months until death. Haematological DLT was defined as WHO grade III neutropenia or thrombocytopenia grade II on day 8, grade IV neutropenia lasting longer than 7 days, febrile neutropenia or thrombocytopenia grade IV at any time during the cycle. Non-haematological DLT was defined as any toxicity exceeding WHO grade II (except alopecia) and nausea and vomiting exceeding WHO grade III. Cardiac toxicity was defined as symptomatic cardiac failure and/or a decrease in LVEF of more than 15% from baseline or more than 10% below the lower normal institutional limit (55%).

*Dose and dose adjustments*

Epirubicin was dissolved in 250 ml 0.9% sodium chloride and given on day 1 as a 30 min intravenous (i.v.) infusion. Vinorelbine was dissolved in 125 ml 0.9% sodium chloride and given on days 1 and 8 as a 10 min i.v. infusion. The interval between courses was 3 weeks with a maximum of six cycles or until disease progression. The starting dose of epirubicin (E) was 100 mg $m^{-2}$ on day 1, and that of vinorelbine (V) 20 mg $m^{-2}$ on days 1 and 8. Level II consisted of E 120 (day 1) and V 20 mg $m^{-2}$ (days 1 and 8), level III: E 120 (day 1)/V (days 1 and 8) 25 mg $m^{-2}$ and level IV: E 135 (day 1)/V 25 mg $m^{-2}$ (days 1 and 8) respectively. Routine prophylaxis for nausea and vomiting (ondansetron chloride $2 \times 8$ mg oral on day 1 and $1 \times 8$ mg oral on day 2, dexamethasone $1 \times 8$ mg i.v. on day 1) was given at all dose levels. At each level at least three patients were entered. If no DLT was found during their first cycle, the following three patients would start at the next dose level. Three additional patients had to be treated at the same dose level if a single patient experienced DLT in the first cycle. If not more than two out of six patients reached DLT, patients were entered into the next dose level. If at least three out of six patients had one (or more) DLT at a certain dose level during the first chemotherapy cycle, the study was finished. This level was defined as the maximum tolerated dose (MTD); the dose one level lower would be the recommended dose for phase II studies. Dose adjustments or treatment delay up to a maximum of 14 days were made for neutrophil and platelet nadir and day 21 counts. Criteria for dose modifications for haematological toxicity are summarised in Table I. The dose of both drugs had to be reduced by 50% if bilirubin was between 35 and 50 $\mu$mol $l^{-1}$ and/or aspartate aminotransferase (ASAT) was between three and five times upper normal limits in the absence of liver metastases. If bilirubin levels were more than 50 $\mu$mol $l^{-1}$ or ASAT more than five times upper normal limits in the absence of liver metastases, patients would go off study. In case of mucositis grade III or IV the dose of both vinorelbine and epirubicin had to be reduced by 25%.

*Response criteria*

Complete response (CR) indicated the disappearance of all known disease, determined by two observations not less than 4 weeks apart. A partial response (PR) was defined as a decrease by 50% or more in the sum of the products of the two largest perpendicular diameters of all measurable lesions, as determined by two consecutive observations not less than 4 weeks apart. A situation in which less than 50% decrease or less than 25% increase in total tumour size occurred was defined as stable disease or no change (NC). Progressive disease (PD) was defined as $\geqslant$25% increase in the size of one or more measurable lesions, or the appearance of new lesions.

### Results

Twenty-one patients were entered into this study: three patients in dose level I and six patients each in dose levels II, III and IV. Their characteristics are summarised in Table II. No DLT was observed in the first dose level. At the second dose level one patient had grade IV neutropenia. The same patient showed WHO grade IV alanine aminotransferase (ALAT) elevation in absence of known liver metastases. Liver function tests returned to normal on day 22. At the third dose level one patient had febrile neutropenia and grade III neutropenia on day 8, another had grade IV neutropenia lasting 8 days. At the fourth dose level, three patients experienced a DLT (grade III neutropenia on day 8 in two patients, and one patient with febrile neutropenia). At this dose level, defined as the MTD, the study was closed. The incidence (median duration, range) of WHO grade IV neutropenia in the first cycle was 33% (one patient, duration 3 days) in level I, 100% in level II (3 days, 1–4 days) and IV (5 days, 1–6 days) and 84% in level III (3 days, 1–6 days). The combination of epirubicin and vinorelbine induces short-lived neutropenia. Myelosuppression was dose related. The median nadirs of leucocytes, neutrophils and platelets are listed in Table III. The median time to nadir counts was 12 days (range 8–15 days). The percentages of cycles associated with haematological toxicity $\geqslant$WHO grade III are shown in Table IV. WHO grade IV leucopenia and neutropenia occurred in 14% and 50% of all cycles respectively. Four patients

**Table I** Criteria for dose modifications for haematological toxicity

| *Day 21 counts* | *Nadir* | *Dose adjustment* |
|---|---|---|
| Neutrophils $\geqslant 1.5 \times 10^9\, l^{-1}$<br>AND<br>Platelets $\geqslant 100 \times 10^9\, l^{-1}$ | Neutrophils $\geqslant 0.2 \times 10^9\, l^{-1}$<br>AND<br>Platelets $\geqslant 50 \times 10^9\, l^{-1}$ | 100% of day 1 of previous cycle (epirubicin and vinorelbine) |
| Neutrophils $\geqslant 1.5 \times 10^9\, l^{-1}$<br>AND<br>Platelets $\geqslant 100 \times 10^9\, l^{-1}$ | Neutrophils $< 0.2 \times 10^9\, l^{-1}$<br>OR<br>Platelets $< 50 \times 10^9\, l^{-1}$<br>OR<br>Febrile neutropenia | Reduce epirubicin and vinorelbine to 75% of day 1 of previous cycle |
| Neutrophils $< 1.5 \times 10^9\, l^{-1}$<br>OR<br>Platelets $< 100 \times 10^9\, l^{-1}$ | Any | Hold therapy. Repeat CBC weekly until neutrophils $\geqslant 1.5 \times 10^9\, l^{-1}$ AND platelets $\geqslant 100 \times 10^9\, l^{-1}$ and then treat patients with dose adjustments according to nadir evaluation. If no recovery is reached after 2 weeks the patient will go off study |

**Table II** Patient characteristics

| | |
|---|---|
| Number of patients | 21 |
| Sex M/F | 17/4 |
| Age (range) years | 53 (36–73) |
| PS (ECOG) | |
| 0 | 6 |
| 1 | 12 |
| 2 | 3 |
| Stage[a] | |
| IIIB | 10 |
| IV | 11 |
| Histology | |
| Squamous | 5 |
| Adeno | 10 |
| Large cell | 6 |
| Weight loss | |
| <10% | 16 |
| >10% | 5 |
| Number of cycles | |
| Median | 4 |
| Total | 84 |
| Reason for discontinuation of treatment | |
| PD | 12 |
| PD and decrease of LVEF | 1 |
| Patient refusal | 2 |
| Toxic deaths | 0 |

[a]According to the American Joint Committee for Cancer Staging (1986).

had to be hospitalised for neutropenic fever for a total of six admissions. Grade IV thrombocytopenia or anaemia was not observed. Dose adjustments were necessary after the first (11 out of 13 dose reductions) and third course (two out of 13 dose reductions respectively) at all dose levels owing to a neutrophil nadir $<0.2 \times 10^9 l^{-1}$ in 11 and neutropenic fever in two courses. At the fourth level one patient received more than one full-dose course, the remaining five patients treated at this dose level had dose reduction. None of the patients required a second dose reduction. Only one course had to be postponed for 1 week owing to insufficient haematological recovery on day 21. Received dose intensity (RDI) in the four levels in mg $m^{-2}$ $week^{-1}$ was E/V 32/6.4 in level I, 37/6.2 in level II, 35/7.2 in level III and E/V 38/7.1 in level IV. RDI/projected dose intensity (PDI) for the ascending dose levels were 0.97, 0.93, 0.87 and 0.85 respectively. Non-haematological toxicity consisted mainly of nausea/vomiting, stomatitis and phlebitis. The percentages of cycles associated with non-haematological toxicity are listed in Table V. Twenty-eight cycles were associated with nausea/vomiting grade I–III despite prophylactic anti-emetic treatment. The median duration of nausea/vomiting was 2.5 days with a range of 1–8 days. From their second or third cycle on, 16 out of 21 patients showed grade I or II phlebitis. Only two of these patients had to be treated with analgesics. Neurotoxicity was not observed. Cardiac toxicity could not be excluded in one patient who complained of fatigue and slight shortness of breath. LVEF measured by MUGA scan decreased from 74% to 50% after four courses corresponding to a total dose of 416 mg $m^{-2}$ epirubicin. This patient eventually died of progressive disease measured on chest radio-

**Table III** Median (range) nadir of blood cell counts

| *Dose* *mg* $m^{-2}$ | *No. of patients/ No. of cycles* | *WBC* $10^9 l^{-1}$ | *Neutrophils* $10^9 l^{-1}$ | *Platelets* $10^9 l^{-1}$ | *Haemoglobin* *g 100* $ml^{-1}$ |
|---|---|---|---|---|---|
| 100/20 | 3/8 | 2.0 (1.2–3.1) | 0.8 (0.2–1.3) | 209 (140–386) | 105 (88–113) |
| 120/20 | 6/30 | 1.3 (0.7–2.3) | 0.2 (0.1–0.6) | 122 (36–199) | 82 (68–113) |
| 120/25 | 6/23 | 1.1 (0.4–2.5) | 0.2 (0.01–0.5) | 128 (87–192) | 89 (76–127) |
| 135/25 | 6/23 | 0.9 (0.2–2.0) | 0.06 (0.02–0.2) | 89 (76–118) | 104 (56–274) |

**Table IV** Cycles (%) with haematological toxicity ≥ WHO III

| *Dose* *(mg* $m^{-2}$*)* | *No. of patients/ no. of cycles* | *Toxicity grade* | *WBC (%)* | *Neutrophils (%)* | *Haemoglobin (%)* | *Platelets (%)* |
|---|---|---|---|---|---|---|
| 100/20 | 3/8 | III | 50 | 25 | 0 | 0 |
| | | IV | 0 | 40 | 0 | 0 |
| 120/20 | 6/30 | III | 27 | 7 | 0 | 3 |
| | | IV | 0 | 30 | 0 | 0 |
| 120/25 | 6/23 | III | 52 | 35 | 0 | 0 |
| | | IV | 17 | 48 | 0 | 0 |
| 135/25 | 6/23 | III | 22 | 9 | 4 | 0 |
| | | IV | 35 | 57 | 0 | 0 |

**Table V** Cycles (%) with non haematological toxicity

| *Dose* *(mg* $m^{-2}$*)* | *No. of patients/ No. of cycles* | *Toxicity grade* | *Nausea/ vomiting (%)* | *Phlebitis (%)* | *Stomatitis (%)* |
|---|---|---|---|---|---|
| 100/20 | 3/8 | I | 38 | 13 | 24 |
| | | II | 0 | 25 | 0 |
| | | III | 13 | 0 | 0 |
| | | IV | 0 | 0 | 0 |
| 120/20 | 6/30 | I | 13 | 23 | 7 |
| | | II | 13 | 7 | 3 |
| | | III | 3 | 0 | 0 |
| | | IV | 0 | 0 | 0 |
| 120/25 | 6/23 | I | 9 | 35 | 0 |
| | | II | 0 | 13 | 9 |
| | | III | 13 | 0 | 0 |
| | | IV | 0 | 0 | 0 |
| 135/25 | 6/23 | I | 30 | 22 | 4 |
| | | II | 13 | 4 | 0 |
| | | III | 0 | 0 | 0 |
| | | IV | 0 | 0 | 0 |

graph, however, without clinical signs of congestive heart failure. Sixteen patients had to be hospitalised during their treatment for a total of 24 admissions. Reasons for hospital admission were red blood cell transfusion (12 patients), neutropenic fever (four patients), pneumonia (one patient) and ischaemic colitis (one patient). Although tumour response was not an end point of this study, we observed two partial responses (one in level II and one in level III), and ten patients with stable disease out of 20 evaluable patients. Response durations were 12 and 4 weeks. Survival of this group showed a range of 3–106 weeks with a median of 21 weeks (with one patient still alive at time of evaluation).

### Discussion

The objective of this study was to determine the MTD of the combination of high-dose epirubicin and vinorelbine in chemotherapy-naive patients with grade IIIB–IV NSCLC. We found neutropenia, neutropenic fever and thrombocytopenia on day 8 as dose-limiting toxicities. In this phase I study we determined the MTD at 135 mg $m^{-2}$ epirubicin (day 1) combined with 25 mg $m^{-2}$ vinorelbine (days 1 and 8). Leucocyte, neutrophil and platelet nadirs occurred on day 12 with dose-related myelosuppression. In only one out of 84 cycles haematological recovery was not complete before day 22. Non-haematological toxicity consisted mainly of nausea and vomiting (in spite of prophylactic anti-emetic treatment), although neither this nor a frequently observed phlebitis of the infusion vein has been a major problem. Cardiac toxicity was possibly encountered in only one out of 21 patients; the decrease in LVEF was not accompanied by clinical signs. Analysis of other high-dose epirubicin studies has shown that high incidences of decreasing LVEF go together with low incidences of congestive heart failure (Feld *et al.*, 1992). Nielsen *et al.* (1990) conclude from their study of 135 epirubicin-treated patients that LVEF is of no predictive value for congestive heart failure, and should only be measured if there is clinical suspicion of cardiac disease. For higher cumulative dosages endomyocardial biopsy may be more indicative for congestive heart failure than LVEF (Torti *et al.*, 1986). A considerable risk of cardiac toxicity was found in patients treated with a cumulative dose of epirubicin above 1000 mg $m^{-2}$ (Shepherd *et al.*, 1989). Therefore, it is questionable whether MUGA scans should be performed routinely in patients who are treated at lower cumulative dosages. In the present study no neurotoxicity and no treatment-related deaths were observed. The MTD for epirubicin and vinorelbine as established in this phase I study differs from an earlier report (Gridelli *et al.*, 1993). They found an MTD of 60 mg $m^{-2}$ epirubicin without granulocyte colony-stimulating factor (G-CSF) and an MTD of 90 mg $m^{-2}$ epirubicin with G-CSF with a fixed dose of 25 mg $m^{-2}$ vinorelbine. Myelosuppression was the dose-limiting toxicity for both treatment groups, as it was in our study. Gridelli *et al.* (1993) defined MTD as the dose that caused myelotoxicity grade III in 50% of cases and grade IV in 20% of cases. In our definition of MTD the duration of the neutropenia was more important than the degree of nadir, which might explain the difference in MTD between these two studies. Myelotoxicity in our study was comparable with that reported for single-agent epirubicin at dosages of 160–180 mg $m^{-2}$ (Feld *et al.*, 1992). From data of this phase I study, we conclude that combination treatment of high-dose epirubicin with vinorelbine causes considerable toxicity. In dose level III only two out of six patients could receive a full-dose second course compared with four out of six patients at dose level II. Also, the RDI in these two levels were almost identical owing to more dose reductions in the higher dose level. Both arguments suggest that dose level II (epirubicin 120 mg $m^{-2}$ combined with vinorelbine 20 mg $m^{-2}$) is the recommended dose for phase II studies. However, as leuco- and neutropenia are the major toxicities of this regimen, a more obvious combination might be high-dose epirubicin with gemcitabine or carboplatin since these drugs cause less leuco- and neutropenia compared with vinorelbine.

### References

BUNN PA. (1991). The role of systemic chemotherapy in non-small cell lung cancer. In: *Current Topics in Lung Cancer*, Bunn PA (ed.) pp. 3–15. Springer: Berlin.

LE CHEVALIER T, BRISGAND D, DOUILLARD JY, PUJOL JL, ALBEROLA V, MONNIER A, RIVIERE A, LIANES P, CHOMY P, CIGOLARI S, GOTTFRIED M, RUFFIE P, PANIZO A, GASPARD MH, RAVAIOLI A, BESENVAL M, BESSON F, MARTINEZ A, BERTHAUD P AND TURSZ T. (1994). Randomized study of vinorelbine and cisplatin *vs* vindesine and cisplatin *vs* vinorelbine alone in advanced non-small-cell lung cancer: results of a European multicenter trial including 612 patients. *J. Clin. Oncol.*, **12**, 360–367.

CVITKOVIC E AND IZZO J. (1992). The current and future place of vinorelbine in cancer chemotherapy. *Drugs*, **44** (suppl 4P), 36–45.

FELD R, WIERZBICKI R, WALDE PLD, SHEPHERD FA, EVANS WK, GUPTA S, SHANNON P AND LASSUS M. (1992). Phase I–II study of high-dose epirubicin and advanced non-small cell lung cancer. *J. Clin. Oncol.*, **10**, 297–303.

GRIDELLI C, DE PLACIDO S, PEPE R, INCORONATO P, AIROMA G, ROSSI A, PALAZZO G AND BIANCO AR. (1993). Phase I study of epirubicin plus vinorelbine with or without G-CSF in advaned non-small cell lung cancer. *Eur. J. Cancer*, **29A**, 1729–1731.

GRILLI R, OXMAN AD AND JULIAN JA. (1993). Chemotherapy for advanced non-small cell lung cancer: how much benefit is enough? *J. Clin. Oncol.*, **11**, 1866–1872.

IHDE DC. (1992). Non-small cell lung cancer. In *Manual of Oncologic Therapeutics*, Wittes RE (ed.) pp. 137–141. Lippincott: Philadelphia.

KRIKORIAN A AND BREILLOUT F. (1991). Vinorelbine (Navelbine). A new semisynthetic vinca alkaloid. *Onkologie*, **14**, 7–12.

LAUNCHBURY AP AND HABBOUBI N. (1993). Epirubicin and doxorubicin: a comparison of their characteristics, therapeutic activity and toxicity. *Cancer Treat. Rev.*, **19**, 197–228.

LILENBAUM RC AND GREEN MR. (1993). Novel chemotherapeutic agents in the treatment of non-small cell lung cancer. *J. Clin. Oncol.*, **11**, 1391–1402.

MARTONI A, MELOTTI B, GUARALDI M AND PANNUTI F. (1991). Activity of high-dose epirubicin in advanced non-small cell lung cancer. *Eur. J. Cancer*, **27**, 1231–1234.

NIELSEN D, JENSEN JB, DOMBERNOVSKY P, MUNCK O, FOGH J, BRYNJOLF I, HAVSTEEN H AND HANSEN M. (1990). Epirubicin cardiotoxicity: a study of patients with advanced breast cancer. *J. Clin. Oncol.*, **8**, 1806–1810.

PLOSKER GL AND FAULDS D. (1993). Epirubicin: A review of its pharmacodynamic and pharmacokinetic properties, and therapeutic use in cancer chemotherapy. *Drugs*, **45**, 788–856.

SHEPHERD FA, FELD R, BLACKSTEIN M, GUPTA S, COOK DJ AND LASSUS M. (1989). Administration of high dose bolus epirubicin (EPI) is not associated with increased cardiotoxicity. *Proc. Am. Soc. Clin. Oncol.*, **8**, 335.

SMIT EF, BERENDSEN HH, PIERS DA, SMEETS J, RIVA A AND POSTMUS PE. (1992). A phase II study of high dose epirubicin in unresectable non-small cell lung cancer. *Br. J. Cancer*, **65**, 405–408.

SORENSEN JB. (1992). Vinorelbine. A review of its antitumour activity in lung cancer. *Drugs*, **44** (suppl 4P), 60–65.

SOUQUET PJ, CHAUVIN F, BOISSEL JP, CELLERINO R, CORMIER Y, GANZ PA, KAASA S, PATER JL, QUOIX E, RAPP E AND TUMARELLO D. (1993). Polychemotherapy in advanced non-small cell lung cancer: a meta-analysis. *Lancet*, **342**, 19–21.

TORTI FM, BRISTOW MM, LUM BL, CARTER SK, HOWES AE, ASTON DA, BROWN BW, HANNIGAN JF, MEYERS FJ, MITCHELL EP AND BILLINGHAM ME. (1986). Cardiotoxicity of epirubicin and doxorubicin: Assessment by endomyocardial biopsy. *Cancer Res.*, **46**, 3722–3727.

VILLAR A, ANTON E AND JIMENO J. (1991). Fractioned high dose (HD) of epirubicin (E) in adenocarcinoma of the lung (ADL). *Lung Cancer*, **7**, 122.

WHO. (1978). *Handbook for Reporting Results of Cancer Treatment.* Nijhoff: The Hague, The Netherlands.

WILS J, UTNAMA I, SALA L, SMEETS J AND RIVA A. (1990). Phase II study of high-dose epirubicin in non-small cell lung cancer. *Eur. J. Cancer*, **26**, 1140–1141.

ZHOU XJ AND RAHMANI R. (1992). Preclinical and clinical pharmacology of vinca alkaloids. *Drugs*, **44** (suppl 4P), 1–16.


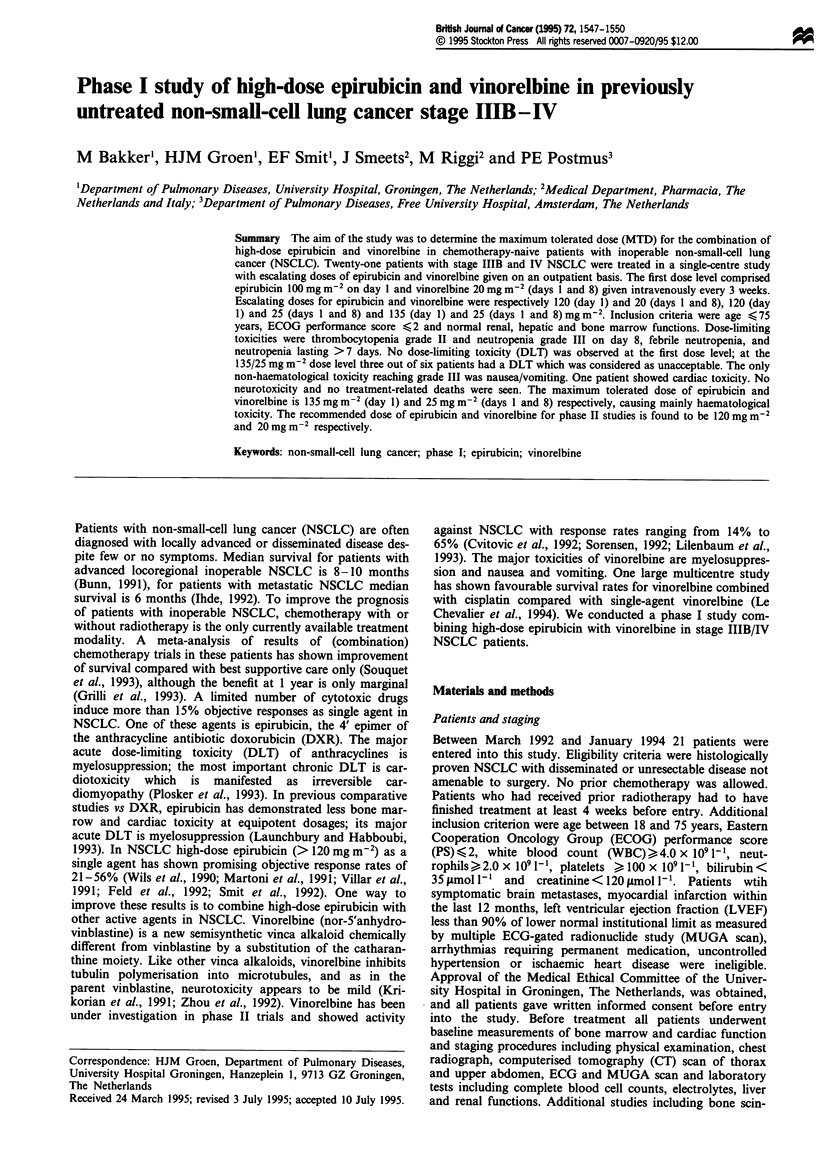

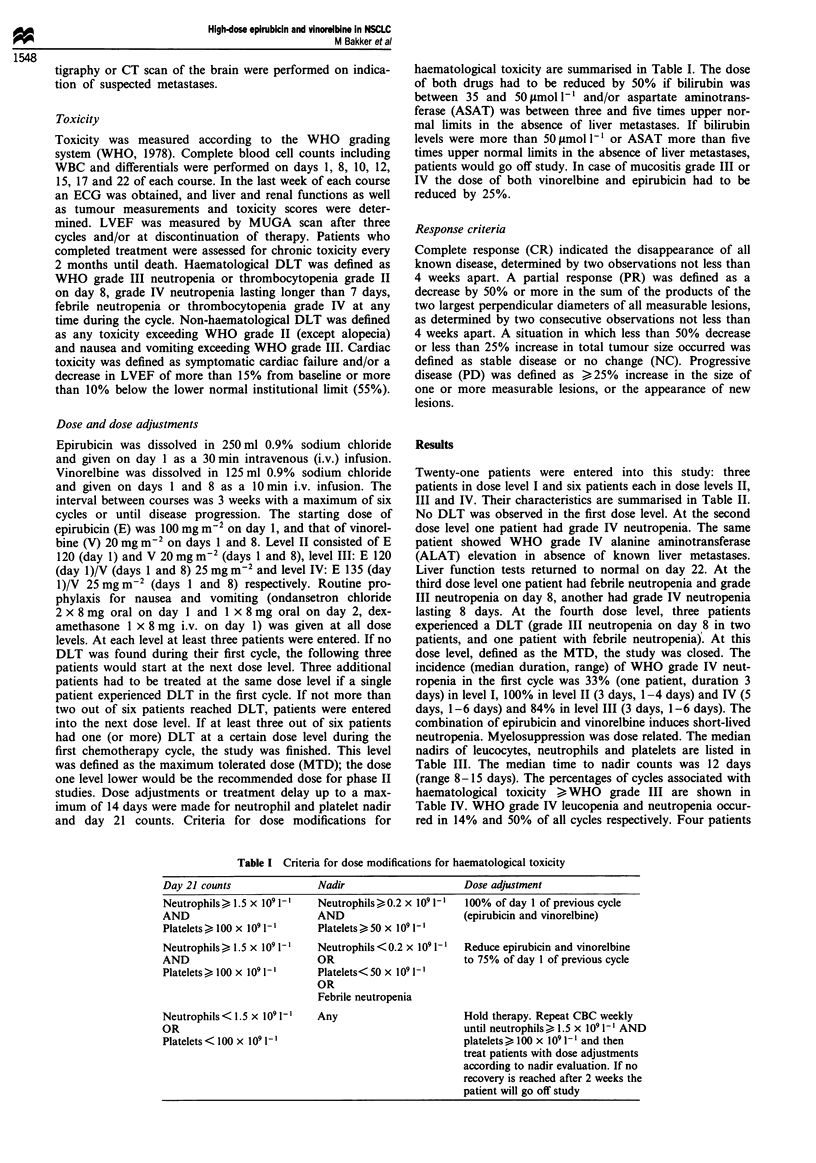

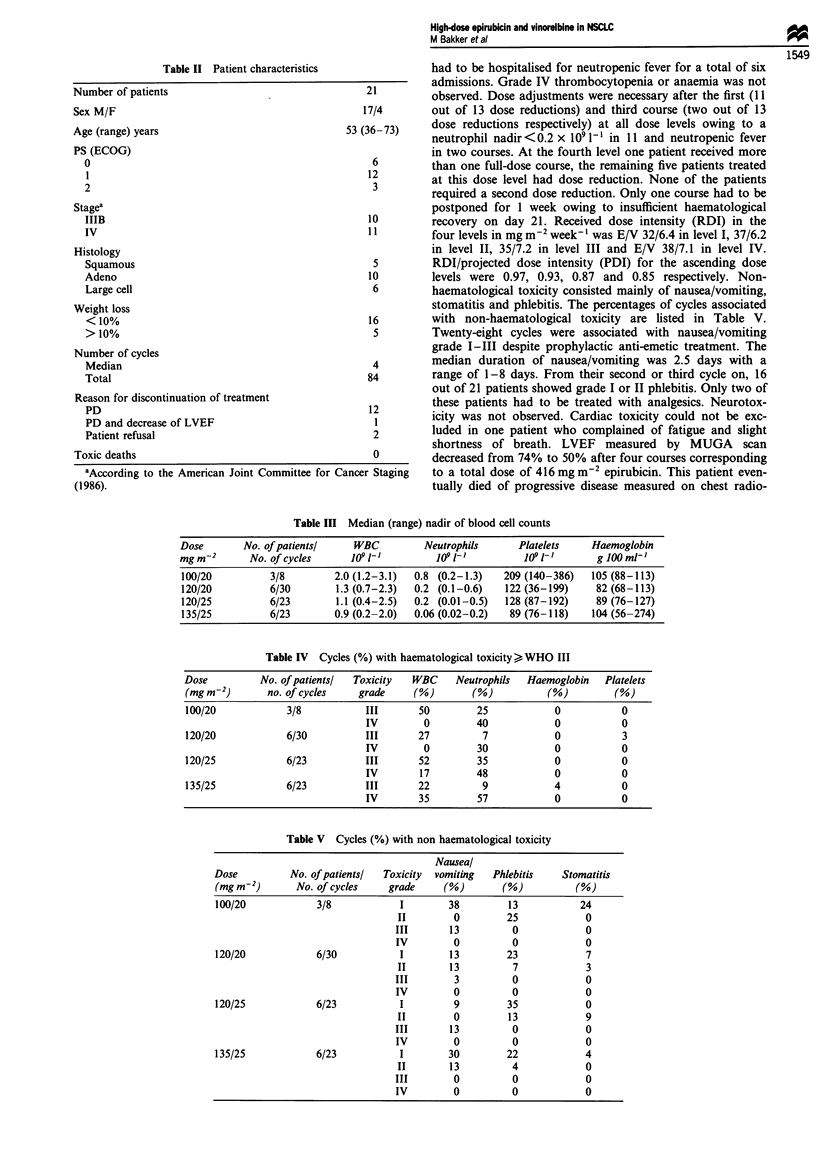

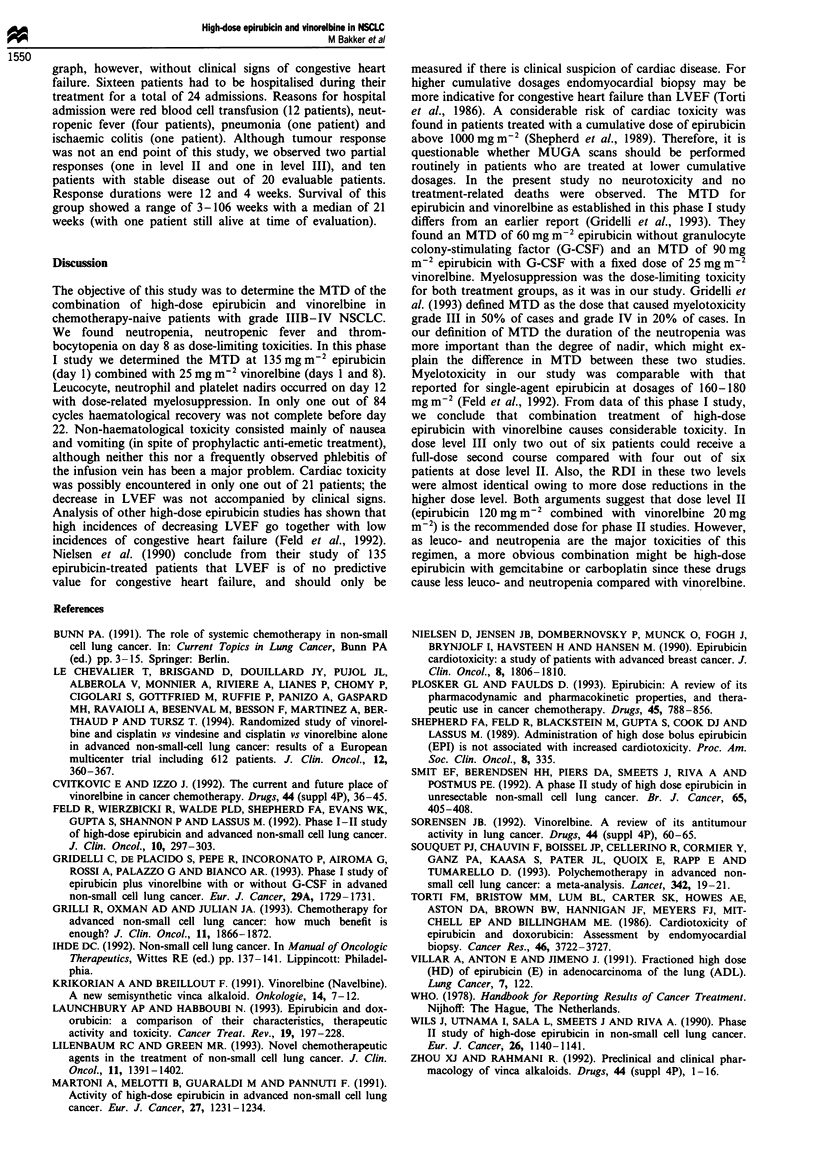


## References

[OCR_00499] Feld R., Wierzbicki R., Walde P. L., Shepherd F. A., Evans W. K., Gupta S., Shannon P., Lassus M. (1992). Phase I-II study of high-dose epirubicin in advanced non-small-cell lung cancer.. J Clin Oncol.

[OCR_00502] Gridelli C., De Placido S., Pepe R., Incoronato P., Airoma G., Rossi A., Palazzolo G., Bianco A. R. (1993). Phase I study of epirubicin plus vinorelbine with or without G-CSF in advanced non-small cell lung cancer.. Eur J Cancer.

[OCR_00508] Grilli R., Oxman A. D., Julian J. A. (1993). Chemotherapy for advanced non-small-cell lung cancer: how much benefit is enough?. J Clin Oncol.

[OCR_00518] Krikorian A., Breillout F. (1991). Vinorelbine (Navelbine). A new semisynthetic vinca alkaloid.. Onkologie.

[OCR_00524] Launchbury A. P., Habboubi N. (1993). Epirubicin and doxorubicin: a comparison of their characteristics, therapeutic activity and toxicity.. Cancer Treat Rev.

[OCR_00482] Le Chevalier T., Brisgand D., Douillard J. Y., Pujol J. L., Alberola V., Monnier A., Riviere A., Lianes P., Chomy P., Cigolari S. (1994). Randomized study of vinorelbine and cisplatin versus vindesine and cisplatin versus vinorelbine alone in advanced non-small-cell lung cancer: results of a European multicenter trial including 612 patients.. J Clin Oncol.

[OCR_00527] Lilenbaum R. C., Green M. R. (1993). Novel chemotherapeutic agents in the treatment of non-small-cell lung cancer.. J Clin Oncol.

[OCR_00532] Martoni A., Melotti B., Guaraldi M., Pannuti F. (1991). Activity of high-dose epirubicin in advanced non-small cell lung cancer.. Eur J Cancer.

[OCR_00540] Nielsen D., Jensen J. B., Dombernowsky P., Munck O., Fogh J., Brynjolf I., Havsteen H., Hansen M. (1990). Epirubicin cardiotoxicity: a study of 135 patients with advanced breast cancer.. J Clin Oncol.

[OCR_00545] Plosker G. L., Faulds D. (1993). Epirubicin. A review of its pharmacodynamic and pharmacokinetic properties, and therapeutic use in cancer chemotherapy.. Drugs.

[OCR_00554] Smit E. F., Berendsen H. H., Piers D. A., Smeets J., Riva A., Postmus P. E. (1992). A phase II study of high dose epirubicin in unresectable non small cell lung cancer.. Br J Cancer.

[OCR_00566] Souquet P. J., Chauvin F., Boissel J. P., Cellerino R., Cormier Y., Ganz P. A., Kaasa S., Pater J. L., Quoix E., Rapp E. (1993). Polychemotherapy in advanced non small cell lung cancer: a meta-analysis.. Lancet.

[OCR_00570] Torti F. M., Bristow M. M., Lum B. L., Carter S. K., Howes A. E., Aston D. A., Brown B. W., Hannigan J. F., Meyers F. J., Mitchell E. P. (1986). Cardiotoxicity of epirubicin and doxorubicin: assessment by endomyocardial biopsy.. Cancer Res.

[OCR_00586] Wils J., Utama I., Sala L., Smeets J., Riva A. (1990). Phase II study of high-dose epirubicin in non-small cell lung cancer.. Eur J Cancer.

